# Composition and Antibacterial Activity of the Essential Oils of *Orthosiphon stamineus* Benth and *Ficus deltoidea* Jack against Pathogenic Oral Bacteria

**DOI:** 10.3390/molecules22122135

**Published:** 2017-12-05

**Authors:** Nuramirah Azizan, Shahida Mohd Said, Zamirah Zainal Abidin, Ibrahim Jantan

**Affiliations:** 1 Drug and Herbal Research Centre, Faculty of Pharmacy, Universiti Kebangsaan Malaysia, Kuala Lumpur 50300, Malaysia; ameera.azizan@gmail.com; 2 Department of Clinical oral Biology, Faculty of Dentistry, Universiti Kebangsaan Malaysia, Kuala Lumpur 50300, Malaysia; zamirah@ukm.edu.my; 3 Department of Periodontology, Faculty of Dentistry, Universiti Kebangsaan Malaysia, Kuala Lumpur 50300, Malaysia; shahidams@ukm.edu.my

**Keywords:** *Orthosiphon stamineus*, *Ficus deltoidea*, essential oils, antibacterial, oral pathogens

## Abstract

In this study, the essential oils of *Orthosiphon stamineus* Benth and *Ficus deltoidea* Jack were evaluated for their antibacterial activity against invasive oral pathogens, namely *Enterococcus faecalis*, *Streptococcus mutans*, *Streptococcus mitis*, *Streptococcus salivarius*, *Aggregatibacter actinomycetemcomitans*, *Porphyromonas gingivalis* and *Fusobacterium nucleatum*. Chemical composition of the oils was analyzed using gas chromatography (GC) and gas chromatography-mass spectrometry (GC-MS). The antibacterial activity of the oils and their major constituents were investigated using the broth microdilution method (minimal inhibitory concentration (MIC) and minimal bactericidal concentration (MBC)). Susceptibility test, anti-adhesion, anti-biofilm, checkerboard and time-kill assays were also carried out. Physiological changes of the bacterial cells after exposure to the oils were observed under the field emission scanning electron microscope (FESEM). *O. stamineus* and *F. deltoidea* oils mainly consisted of sesquiterpenoids (44.6% and 60.9%, respectively), and β-caryophyllene was the most abundant compound in both oils (26.3% and 36.3%, respectively). Other compounds present in *O. stamineus* were α-humulene (5.1%) and eugenol (8.1%), while α-humulene (5.5%) and germacrene D (7.7%) were dominant in *F. deltoidea*. The oils of both plants showed moderate to strong inhibition against all tested bacteria with MIC and MBC values ranging 0.63–2.5 mg/mL. However, none showed any inhibition on monospecies biofilms. The time-kill assay showed that combination of both oils with amoxicillin at concentrations of 1× and 2× MIC values demonstrated additive antibacterial effect. The FESEM study showed that both oils produced significant alterations on the cells of Gram-negative bacteria as they became pleomorphic and lysed. In conclusion, the study indicated that the oils of *O. stamineus* and *F. deltoidea* possessed moderate to strong antibacterial properties against the seven strains pathogenic oral bacteria and may have caused disturbances of membrane structure or cell wall of the bacteria.

## 1. Introduction

Oral health problems, particularly periodontal diseases, dental caries and endodontic infections, are the most significant destructive processes in the oral cavity and are a costly burden to the public globally. Periodontal diseases are pathologic conditions of bacterial infection of the structures around the teeth (including the gums, the cementum that covers the root, the periodontal ligament and the alveolar bone) that can lead to tooth loss affecting more than half of all adults. Dental caries (tooth decay or cavities) are the most common and widespread chronic oral diseases that affect children and adults. They are irreversible infectious diseases of the teeth leading to cavities in the teeth structure, thus compromising the structure and function of the teeth [1,2]. Meanwhile, endodontic infections are polymicrobial infections that affect the tooth canal system which allow the teeth to grow, mature and respond to stress. Oral diseases are initiated by bacterial infection in the oral cavity and trigger inflammatory responses that will continue until the source of infection is removed through dental treatment of choice [3,4]. The combined ill effects of these major oral diseases weaken bodily defense and may also serve as portal of entry to other more chronic and opportunistic infections. Indeed, oral diseases create a silent epidemic, placing a heavy burden on worldwide populations [5].

Previous studies have indicated that clinicians were not intervening early enough with diagnosis or treatment. Despite some improvements, a high prevalence of dental caries, periodontal diseases and endodontic infections are still recorded worldwide due to the complications caused by emergence of antibiotics resistance, treatment failure and poor oral hygiene. In addition, in recent years, oral disease has also been linked to other health problems such as cardiovascular diseases and diabetic complication, and associates the mouth as a potential source for bacteria to spread to the rest of the body [6,7]. These concerns are being used to find other possible alternatives to efficiently manage destructive oral diseases and ultimately provide better evidence-based management of oral disease.

Herbal essential oils such as cinnamon and tea tree oils have been traditionally used for oral care purposes. The benefits also come from their variety of pharmacological properties including antimicrobial, anti-diabetic, antioxidants, treatment for cancer and cardiovascular diseases. Oils from these herbs are rapidly growing in popularity because they act as natural medicine and have been proven to have beneficial healing power with little side effects [8,9,10]. *Orthosiphon stamineus* Benth, locally known as misai kucing or kumis kucing in Malaysia, is a medicinal herb belonging to the Labiatae or Lamiaceae family. The name is given due to the long filaments that look like cat whiskers, which is one of the unique morphological features of the flowers. This valuable herbaceous woody plant can be found in regions with tropical climate [11]. It has been proven that its extract effectively treated various ailments as it is believed to have various pharmacological properties such as anti-allergic, antihypertensive, diuretic, antioxidant, antidiabetic, anti-inflammatory, mild antiseptic, antifungal and even exhibited considerable antibacterial and antiviral activities [12]. *Ficus deltoidea* Jack or mistletoe fog, which is also known as mas cotek among Malaysians, is a plant of the family Moraceae. Ethnobotanical approaches have claimed its medicinal potential such as to treat diabetes, rheumatism, cold, sores, toothache, headache and other ailments. Additionally, it is also being used traditionally for general health benefits such as as a tonic for reproduction system. Scientific studies have found that these plants have a wide range of beneficial phytochemicals and exhibit various pharmacological activities such as anti-inflammatory, antidiabetic, anticancer, antifungal and antimicrobial [13,14,15]. However, there is still limited information regarding the effects of the essential oils of both herbs on the health and well-being of the oral cavity.

This study focused on assessing the antibacterial properties as well as profiling the bioactive compounds of *O. stamineus* and *F. deltoidea* essential oils, with a high expectation to create and develop a highly effective intervention against invasive oral infections caused by different types of oral pathogens. Besides, answering these questions will also allow us to gain better insight of the significant contribution of *O. stamineus* and *F. deltoidea* towards combating selected oral pathogens. This latter may pave the way to pharmaceutical applications in the near future and consequently result in the development of a novel preventive strategy to prevent the infections caused by these pathogens.

## 2. Results

### 2.1. Analysis of the Components of the Essential Oils

Hydro-distillation of the whole parts of the plants yielded 0.33% of *O. stamineus* oil and 0.37% of *F. deltoidea* oil, based on a dry weight. The GC analysis of the oils resulted in identification of 30 and 40 compounds for *O. stamineus* and *F. deltoidea*, respectively. The quantitative and qualitative data of the volatile components of both oils are shown in Table 1, in order of elution on a DB-5 type column. Our study showed that *O. stamineus* oil contained mainly β-caryophyllene (26.31%), bicyclogermacrene (7.7%), methyl eugenol (7.4%), α-humulene (5.06%), α-copaene (2.26%) and naphthalene (1.13), whereas the major constituents of *F. deltoidea* oil were β-caryophyllene (36.3%), eugenol (8.1%), germacrene D (7.7%), α-humulene (5.5%) and α-copaene (3.3%).

### 2.2. Antibacterial Activity of the Essential Oils and Their Major Compounds

The antibacterial activity of the oils and their major compounds against the seven species of pathogenic oral bacteria are shown in Table 2. The results indicated that there was no different in the antibacterial activity of *O. stamineus* and *F. deltoidea* oils against all Gram-positive and Gram-negative bacteria. In addition, the antibacterial activity of the essential oil standards (*trans*-caryophyllene, α-humulene, eugenol and germacrene D) was greater against Gram-negative bacteria as compared to Gram-positive bacteria. On the other hand, all of the monospecies biofilms of the oral bacteria tested were found resistant against both oils and the essential oil standards at the tested range of 0.04–5.0 mg/mL. Our study also showed that the aerobic bacteria, namely *Enterococcus faecalis*, *Streptococcus mutans*, *S. mitis* and *S. salivarius*, were moderately susceptible to all the oils and the essential oil standards. However, the anaerobic Gram-negative bacteria, *Aggregatibacter actinomycetemcomitans*, *Porphyromonas gingivalis* and *Fusobacterium nucleatum*, were slightly susceptible to the essential oil standards. Nevertheless, the antibacterial activity of both oils and the standards was still at the intermediate level as compared to the activity of amoxicillin (positive control), i.e., MIC ≤ 8 µg/mL is considered susceptible and MIC ≥ 16 µg/mL is resistant for Gram-positive bacteria, whereas MIC ≤ 0.5 µg/mL is considered susceptible and MIC ≥2 µg/mL is resistant for Gram-negative bacteria [16].

### 2.3. Morphological Changes of the Bacterial Cells

Any modification or alteration on the morphology of the bacterial cells after being treated with the oils and amoxicillin were observed under the field emission scan electron microscope (FESEM). The oral bacteria were treated with the oils at their respective MIC values and results are shown in Table 3 and Table 4. There were noteworthy alterations on the bacterial cells with regards to its shape, size and arrangement. The arrangement of the bacterial cells was still intact in clusters, while the morphology of the bacterial cells was observed slightly altered to become rough and irregular when compared to the controls (untreated bacteria). Generally, treatment with *O. stamineus* and *F. deltoidea* oils gave similar effect on all tested oral Gram-positive and Gram-negative bacteria. Comparison with normal bacterial cells showed that the cells became pleomorphic, irregular in size and some ruptured. The bacterial cells also presented irregular arrangement as they merged and attached to each other. Few cells appeared collapsed and lysed, causing possibly leakage of the intracellular contents. These observations suggest that the mechanism of antibacterial activity of both oils may be related to the disturbance of membrane structure or cell wall of the bacteria upon exposure [17,18]. Meanwhile, tested Gram-negative bacteria showed drastic morphological alterations upon treatment with amoxicillin as compared to the tested oral Gram-positive bacteria. It is possible that the inhibition and interference with synthesis of cell wall may have caused the tested Gram-negative bacterial cells to lose control over its shape and size and further disruption in homeostasis leads to shrinkage and eventually death of those cells.

### 2.4. Herbal Oils and Amoxicillin Synergistic Effect

**Figure 1:**
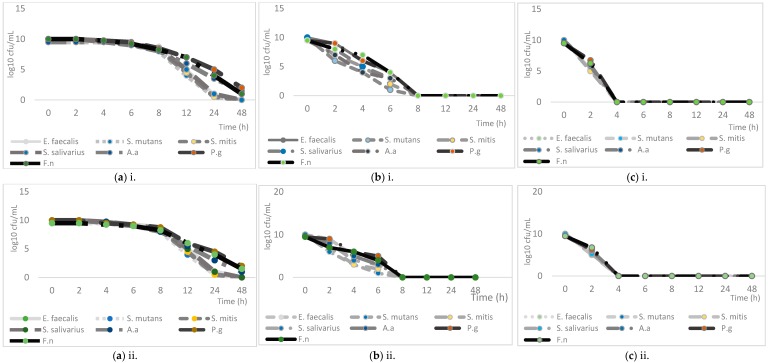
Time-kill curves of the antibacterial activity of the combination of both oils and amoxicillin (i: *O. stamineus;* and ii: *F. deltoidea)* at: (**a**) 0.5 MIC concentration of combination; (**b**) 1× MIC concentration of combination; and (**c**) 2× MIC concentration of combination.

The synergism activity of the essential oils combined with amoxicillin as determined by the checkerboard microdilution technique is presented in Table 5. The combination of both oils at their MIC value (0.63 to 1.25 mg/mL) and also the combination of *O. stamineus* and *F. deltoidea* oils with amoxicillin tested at their MIC ranges (0.63 to 1.25 mg/mL) yielded additive effect as defined by the FIC index of 0.75. An additive effect is observed when there is two-fold decreasing in the MIC of both components and thus the effect of the combination is equal to the sum of the effect of individual component [19,20]. Time-kill experiment was performed to confirm and validate the activity of the combination of both essential oils with amoxicillin. All combinations of specified concentration showed bacteriostatic and bactericidal effect in a time- and dose-dependent manner (Figure 1). The most effective combination was the 2× MIC as it consisted of the highest concentration of each agent in the combination. The bactericidal endpoints for all the oral bacteria tested were reached after 8 and 4 h of incubation at concentrations of 1× and 2× MIC of the combination, respectively.

## 3. Discussion

The essential oils of *O. stamineus* and *F. deltoidea* were mainly made up of sesquiterpenoids (44.6% and 60.9%, respectively). Both oils were also rich in monoterpenoids and phenylpropanoids. Other major compounds present in *O. stamineus* oil were α-humulene, eugenol, bicyclogermacrene and methyl eugenol, while α-humulene, germacrene D and eugenol were dominant in *F. deltoidea* oil. Previous study by Hossain et al. also stated that terpenoids were the major components of *O. stamineus* oil, while studies reported by Grison-Pige et al. showed that the major compounds present in the essential oils of *Ficus* sp. were sesquiterpenoids [21,22,23].

Amoxicillin, a semisynthetic drug belonging to the class penicillin, is a β-lactam antibiotic. It has a broad bactericidal spectrum against both Gram-positive and Gram-negative bacteria. Its cytotoxicity involves the inhibition of biosynthesis and repair of the bacterial mucopeptide cell wall through prevention of peptidoglycan crosslinking by inhibiting the peptidoglycan transpeptidase which consequently lead to the lysis of the bacterial cells (also known as cell wall inhibitor). However, a few studies revealed that the susceptibility of Gram-negative bacteria was greater than Gram-positive bacteria. This is due to its more rapid penetration ability against the cell wall of Gram-negative bacteria [24,25].

The essential oils standards tested in this study, caryophyllene, humulene and eugenol were the major compounds present in the tested oils. The essential oils of both plants and essential oil standards exhibited potential bactericidal effects against the tested oral pathogenic bacteria. Although the mode of action is still not fully understood, it is likely that the mechanism is related to the destruction of cellular integrity involving disruption of membrane structure and functions. One possible scenario is the irreversible damage to the membrane of bacterial cells, which induce losses and leakage of cellular contents, leading directly to the lysis of bacteria and therefore to its death. Moreover, the antibacterial activity may also be influenced by the susceptibility of the bacterial cells towards the chemical composition of the essential oils [26,27,28,29]. β-Caryophyllene is a natural bicyclic sesquiterpene, a unique compound made of three isoprene units and a cyclobutane ring. It is commonly found as a mixture in aromatic plants together with α-humulene and isocaryophyllene [30]. α-Humulene, a naturally occurring monocyclic sesquiterpene made of three isoprene units, but does not contain a cyclobutane ring. It is an isomer of β-caryophyllene. Germacrene D is also a sesquiterpene presents in plants. Scientifically, terpenes have been shown to cause damage to the morphological structure of bacterial cell membrane. Eugenol is a well-known natural product of phenylpropene. The antibacterial activity of all these compounds has been reported in previous studies although not against oral bacteria [31,32,33]. The possible mechanisms of antibacterial activity include disruption of the membrane and wall of bacteria cells resulting in discharge of the intracellular components. Thus, these bioactive compounds might attribute to the antibacterial activity of *O. stamineus* and *F. deltoidea* oils against the oral pathogens.

It is noteworthy that the antibacterial properties may also be contributed by other minor compounds present in the essential oils, as well as due to the possible interactions between the minor compounds or with the major active compounds. Mode of actions of the antibacterial activity may differ considerably depending on their interactive functions. Therefore, synergistic and/or additive effects might be taken into account and it is likely that the inhibition effect involve several sites of action at the cellular level due to a single and/or several different mechanism(s) of action of one or several compounds. Moreover, association of essential oil with antibiotics may lead to exertion of different mechanism of action thus provide new choices in overcoming the unsolved crisis of bacterial resistance [34]. The minor components that have been reported to possess antibacterial potential present in *O. stamineus* and *F. deltoidea* oils include linalool [35,36], caryophyllene oxide, α-pinene and β-pinene [37,38,39]. Studies done by Sikkema et al. revealed that loss of membrane integrity was detected in the presence of cyclic terpenes such as α-pinene and β-pinene. Furthermore, aromatic compounds such as cinnamaldehyde was shown to inhibit the growth and impaired the physiology of the bacterial cells through its action on the bacterial cytoplasmic membrane [40,41].

It was often reported that Gram-negative bacteria are less susceptible to the oils present in plants [42]. As compared to the Gram-positive bacteria, Gram-negative bacteria have more complex cell wall notably due to the presence of membrane located outside of the peptidoglycan layers, known as an outer membrane. It is constituted mainly with lipopolysaccharide (LPS) that acts as a molecular filter or barrier to the hydrophilic compounds which may be one of the reason for the higher resistance of the Gram-negative bacteria. This difference also could be due to the variation in the rate and ability of penetration or disruptive capability of the essential oil compounds through the bacterial cell wall and cell membrane. However, in our study, all of the Gram-positive and Gram-negative bacteria showed almost similar pattern of susceptibility against both essential oils and essential oil standards. Interestingly, the greater antibacterial effects of both essential oils against Gram-negative bacteria were clearly demonstrated from the MIC and MBC assays, and SEM analysis. Nevertheless, from the time-kill curve analysis, it clearly shows that the Gram-positive bacteria were indeed more susceptible as shorter time was needed for the combination to have an effect on the Gram-positive bacteria as compared to the Gram-negative bacteria. This is in accordance to a study on the effects of a variety of essential oil components towards the outer membrane permeability in Gram-negative bacteria and Gram-positive bacteria [43].

Essential oil is a concentrated scented source of phytochemicals that offer many therapeutic benefits. Essential oil uses vary, ranging from health care products, aromatherapy, alternative medicine, household cleaning, and personal beauty care products to oral care products such as toothpastes and mouthwashes. Apart from the antimicrobial and antiseptic activities against oral pathogens, essential oils are also very helpful in the management of oral malodor and promoting healthy gums. Moreover, whitening ability and nice fragrant of essential oils add on its unique characteristics in addition to pain relief properties. Peppermint, clove, cinnamon, rosemary and tea tree are some of the well known plants with essential oils that have those beneficial properties and have been added as ingredients in a few oral care products commercially or traditionally. Besides, oral care products containing essential oil have also been proven as safe to be used in either healthy or unhealthy individuals [44,45,46,47,48]. Therefore, essential oils are one of the best alternative candidates in replacing the chemical or artificial flavoring, coloring and preservatives present in current oral care products.

## 4. Materials and Methods

### 4.1. Chemicals and Reagents

Amoxicillin and phosphate buffered saline were purchased from Sigma (Sigma-Aldrich, St. Louis, MO, USA). All types of microbiological culture media were purchased from Oxoid (Oxoid Ltd., Cheshire, UK). *Trans*-caryophyllene, α-humulene, eugenol and germacrene D were purchased from Chromadex (ChromaDex, Irvine, CA, USA). Tween 20, defibrinated horse blood, dimethyl sulfoxide (DMSO), absolute ethanol, ethyl acetate, methanol, acetonitrile, formic acid, and water (HPLC grade) were purchased from Merck (Merck KGaA, Darmstadt, Germany).

### 4.2. Plant Materials and Preparation of Oils

Fresh whole plants of *O. stamineus* and *F. deltoidea* were collected from Alor Star, Kedah, Malaysia in February 2015 and deposited as voucher specimens in the Universiti Kebangsaan Malaysia (UKM) Herbarium (voucher Nos.: 4021 and 40224, respectively). Stems and leaves of the plants were left to dry under shades for approximately one week, grounded and then weighed. The oils were extracted using hydro-distillation method with Clevenger’s apparatus (Sigma Aldrich, St. Louis, MO, USA). One hundred grams of powdered sample were dissolved in distilled water for 8 h and eventually the oily layers were separated and dried over anhydrous sodium sulfate. Oil yields were calculated based on the dry weight of plant and averaged for three experiments.

### 4.3. Gas Chromatographic (GC) Analyses of the Essential Oils

Analysis of the oils was first carried out using the Shimadzu GC2000 with column DB-5 (1 µm thickness, 30.0 m length, 0.25 mm diameter) with the temperature of injector and detector maintained at 250 °C initially. Oils were dissolved in ethyl acetate and 0.1 µL was injected in individual mode. Nitrogen was used as the carrier gas with a flow rate of 1.0 mL/min. Temperature of the oven was programmed at 75 °C at the initial step for 10 min, and then gradual increased to 230 °C at 3 °C/min for 5 min. A homologous series of n-alkane standards (C_9_ to C_22_) were also analyzed under similar condition as used for the oils. Kovat indices of the individual components of the oils were later calculated relative to the n-alkanes.

Additionally, the oils were analyzed using gas chromatography–mass spectrometry (GC-MS) (Agilent technologies, Santa Clara, CA, USA) using Agilent 7890A and Agilent 5975C inert MSD (70 eV direct inlet) equipped with triple-axis detector and SE-30 (30 m × 0.25 mm; film thickness, 0.25 μm) column. Nitrogen gas was used as the carrier gas at a linear velocity of 50 cm^3^/min. The oven temperature was programmed at 60 °C for 10 min and increased gradually to 230 °C at 3 °C/min. The MSD Chemstation was used to detect all the peaks in the raw GC chromatogram and identification of all peaks was made using NIST/EPA/NIH version 2.0 database (Agilent technologies). Components were also identified by comparing their relative retention indices with those in the literature [49].

### 4.4. Bacterial Culture and Maintenance of Growth

Oral Gram-positive facultative anaerobes (*Enterococcus faecalis* ATCC 29212, *Streptococcus mutans* ATCC 25175, *S. mitis* ATCC 6249 and *S. salivarius* ATCC 13419) and Gram-negative obligate anaerobes (*Aggregatibacter actinomycetemcomitans* ATCC 29522, *Porphyromonas gingivalis* ATCC 33277, and *Fusobacterium nucleatum* ATCC 25586) were used in this study. *E. faecalis* was grown on brain heart infusion agar (BHI) while Streptococci were grown on mitis salivarius agar (MSA). Blood agar supplemented with hemin, vitamin K (menadione) and cysteine was used to isolate *P. gingivalis* and blood agar supplemented with yeast, hemin, vitamin K (menadione) and cysteine was used to isolate *F. nucleatum* whereas trypticase soy agar supplemented with yeast extract, horse serum, vancomycin and bacitracin (TSBV) was used to isolate *A. actinomycetemcomitans*. The procedure was in accordance to standard procedure recommended by guidelines from the Clinical and Laboratory Standards Institute 2004 (CLSI) with a few modifications [50].

### 4.5. Determination of Minimum Inhibitory Concentration (MIC) and Minimum Bactericidal Concentration (MBC)

The broth microdilution technique was used to determine the MIC values of each oil and oil standards (>98% purity) namely β-caryophyllene, α-humulene, eugenol and germacrene D (Chromadex), in a 96-wells microtiter plates (CLSI) [51,52,53]. Initially, inoculum of bacteria in suspension media (10^5^ cfu/mL) was exposed to the oils prepared at a series of concentration range (10–0.08 mg/mL in two-fold serial), followed by 24–48 h of incubation period either in aerobic or anaerobic condition accordingly. The bacterial suspension in broth and the test samples in broth were used as negative controls to ensure medium sterility while amoxicillin (0.1 mg/mL prepared in sterile filtered water) acted as positive control.

The MIC value was confirmed visually by determining the lowest concentration of the test sample that inhibited visible growth of bacteria and also by reading of absorbance at 590 nm wavelength using Varioscan plate reader (Fisher Scientific, Hampton, NH, USA). To determine the MBC value, 10 µL of bacterial inoculum from each well of the determined MIC starting from the MIC value to the highest concentration in the series, were transferred and cultured on agar plates (BHI or BHI-T) and incubated for another 24–48 h. The MBC value is the lowest concentration of the test sample that able to kill the bacteria and thus showed no bacterial growth on the agar. All assays were carried out in triplicates in three independent experiments.

### 4.6. Checkerboard Assay and Time-Kill Assay

The checkerboard assay determined the synergistic antibacterial effect of combination between both oils and the oil with an antibiotic (Amoxicillin) [19,54,55]. The evaluation was carried out by using broth microdilution (two-fold) method in a 96-well microtiter plate that already being inoculated with the tested oral pathogens. Combination of essential oils and amoxicillin (1/8, ¼, ½, 1 and 2) was prepared accordingly. All assays were carried out in triplicates in three independent experiments. Synergism is defined as the fractional inhibitory concentration (FIC) and the indices were calculated using the formula below:

(i) The FIC of oil (FIC_A_) = MIC combination/MIC alone.
(ii) The FIC of antibiotic (FIC_B_) = MIC combination/MIC alone.
(iii) FIC index = FIC_A_ + FIC_B_ (FIC index of the combination in each well is the sum of the FIC for each drug (test sample and antibiotic) present in the well).

Synergism is defined as an FIC index ≤ 0.5; additive effect is defined as an FIC index > 0.5 and ≤ 1; indifference effect is defined as an FIC index > 1 and ≤ 2; and antagonism effect is defined as an FIC index > 4.

Time-kill assay was carried out to evaluate the bactericidal activity of the respective combination oils-antibiotics against the tested oral pathogens. The duration of the bacteriostatic and bactericidal effect can be determined by plotting the number of viable bacterial colonies remaining after exposure with the combination. The cultures of all pathogenic oral bacteria tested with a cell density of 10^5^ cfu/mL were exposed to the combination (essential oil + Amoxicillin) at their ½, 1× and also 2× MIC concentration at different time intervals (0, 2, 4, 6, 8, 12, 24 h). Time-kill curve was plotted as log10 cfu/mL vs time functions to illustrate the relationship between the two variables. All assays were carried out in triplicates in three independent experiments.

### 4.7. Anti-Adhesion and Anti-Biofilm Assays

The anti-adhesion assay assessed the effect of the oils and the essential oil standards on the adhesion ability of *E. faecalis*, *A. actinomycetemcomitans*, *P. gingivalis* and *F. nucleatum* at 12th, 24th, 48th and 72nd hour of incubation period at 37 °C in aerobic or anaerobic condition. An aliquot of bacterial suspension of 10^5^ cfu/mL were dispensed in a 96-well microtiter plate containing the oils or the essential oil standards at 1:1 ratio. Untreated cells acted as negative control whereas amoxicillin-treated cells (5.0 mg/mL in DMSO) acted as positive control. After the incubation, wells were washed with distilled water to remove non-adhered bacteria. Quantification of the remaining adhered bacteria was done by adding 0.1% crystal violet dye into each well and incubated at room temperature for 15 min before being washed for three times with 200 µL of sterile distilled water. Then, bacteria in each well was fixed with 100 µL of 95% ethanol (95% ethanol in water) for 10 min. The crystal violet stained cells were extracted and their optical density (OD) were measured at 590 nm wavelength. All assays were carried out in triplicates in three independent experiments 

The anti-biofilm assay determined the effect of the oils and the essential oil standards on the pre-formed biofilm which will suggest the oils and their active compounds ability to remove by possible killing bacteria in biofilm. A 72 h preformed monospecies biofilm for each *E. faecalis*, *A. actinomycetemcomitans*, *P. gingivalis* and *F. nucleatum* was developed in a 96-well microtitre plate incubated at 37 °C anaerobically. The oils and the essential oil standards were then added into the pre-formed biofilm and further incubated accordingly, either aerobically or anaerobically. Untreated pre-formed biofilm acted as negative control, whereas amoxicillin-treated biofilm (5.0 mg/mL in DMSO) acted as positive control. The density of untreated and treated biofilms was quantified by using 0.1% crystal violet staining procedure as described above [56,57,58]. All assays were carried out in triplicates in three independent experiments.

### 4.8. Observation under the Field Emission Scanning Electron Microscope (FESEM)

This method was modified from Zainal-Abidin et al. [59] and standard operating protocol adapted by the Electron Microscopy Unit, Universiti Kebangsaan Malaysia. Bacterial suspensions after 24–48 h incubation period were adjusted to the final concentration of 10^5^ cfu/mL before being exposed to the oils and the essential oil standards at their MIC concentration for 4 h. Then, the bacteria were harvested, washed with PBS and fixed in 2% glutaraldehyde (2% glutaraldehyde in PBS). The bacteria also underwent dehydration with a series of increasing concentration of ethanol (50%, 75%, 85%, 95% and 100%), treatment, critical-point drying and gold coating before observation under the FESEM to observe changes on the bacterial morphological feature upon exposure to the essential oil.

## 5. Conclusions

The essential oils of *O. stamineus* Benth and *F. deltoidea* Jack were rich in sesquiterpenes, monoterpenes and phenylpropanoids. Both essential oils possessed bacteriostatic and bactericidal activities against oral Gram-positive and Gram-negative bacteria, which may be contributed by the presence of their active constituents, such as *trans*-caryophyllene, α-humulene, eugenol and germacrene D. These findings suggest the potential benefit of using both oils in the management of oral infectious diseases and as new alternative antibacterial agents to substitute synthetic antimicrobials.

## Figures and Tables

**Table 1:** Percentage composition of *O. stamineus* and *F. deltoidea* essential oils.

No.	Compound	*O. stamineus*	*F. deltoidea*	Kovat Index Value	Methods of Identification
		Percentage Composition	Percentage Composition		
**1**	α-Pinene	0.1	-	939	a, b
**2**	Camphene	0.05	-	954	a, b
**3**	1-Octen-3-ol	0.35	-	979	a, b
**4**	1,3-Benzenediamine	0.1	-	1169	a, b
**5**	Naphthalene	3.8	-	1181	a, b
**6**	*trans*-Geraniol	0.3	-	1253	a, b
**7**	Anethole	1.1	-	1253	a, b
**8**	Cinnamaldehyde	0.4	-	1270	a, b
**9**	Bornyl acetate	0.16	-	1292	a, b
**10**	1-Methylnaphthalene	0.55	-	1289	a, b
**11**	2-Methylnaphthalene	0.5	-	1312	a, b
**12**	α-Cubebene	0.98	1.55	1351	a, b
**13**	Eugenol	8.1	-	1359	a, b
**14**	α-Copaene	2.26	3.3	1377	a, b
**15**	β-Bourbonene	0.57	0.09	1388	a, b
**16**	Methyl eugenol	7.4	-	1392	a, b
**17**	7-Tetradecane	0.34	-	1400	a, b
**18**	β-Caryophyllene	26.31	36.3	1409	a, b
**19**	E-β-Damascenone	0.6	-	1414	a, b
**20**	γ-Elemene	0.25	-	1437	a, b
**21**	Aromadendrene	0.9	-	1441	a, b
**22**	α-Humulene	5.06	5.5	1455	a, b
**23**	γ-Muurolene	0.2	0.39	1480	a, b
**24**	Bicyclogermacrene	7.68	-	1500	a, b
**25**	β-Bisabolene	0.4	-	1506	a, b
**26**	*cis*-Z-α-Bisabolene epoxide	0.4	-	1507	a, b
**27**	*trans*-Calamenene	0.5	-	1529	a, b
**28**	α-Calacorene	0.6	-	1546	a, b
**29**	Dimethyl-ionone	0.67	-	1567	a, b
**30**	Caryophyllene oxide	1.2	8.7	1583	a, b
**31**	(Z)-β-Ocimene	-	0.1	1037	a, b
**32**	Linalool	-	0.4	1097	a, b
**33**	α-Ylangene	-	0.14	1375	a, b
**34**	β-Elemene	-	0.1	1391	a, b
**35**	α-Santalene	-	0.1	1418	a, b
**36**	β-Copaene	-	0.3	1432	a, b
**37**	α-*trans*-Bergamotene	-	0.4	1435	a, b
**38**	β-Farnesene	-	0.5	1457	a, b
**39**	Alloaromadandrene	-	1.5	1460	a, b
**40**	Germacrene D	-	7.68	1485	a, b
**41**	β-Selinene	-	1.1	1490	a, b
**42**	α-Selinene	-	1.03	1498	a, b
**43**	α-Muurolene	-	0.5	1500	a, b
**44**	α-Farnesene	-	1.1	1506	a, b
**45**	Germacrene A	-	1.0	1509	a, b
**46**	Myristicin	-	1.7	1519	a, b
**47**	δ-Cadinene	-	1.3	1523	a, b
**48**	α-Cadinene	-	1.6	1539	a, b
**49**	Germacrene B	-	1.5	1561	a, b
**50**	Dendrolasin	-	0.7	1572	a, b
**51**	Himachalene epoxide	-	0.1	1580	a, b
**52**	*cis*-β-Elemonene	-	0.2	1590	a, b
**53**	Humulene epoxide	-	0.1	1608	a, b
**54**	β-Himachalene oxide	-	1.4	1616	a, b
**55**	α-Muurolol	-	0.3	1646	a, b
**56**	α-Cadinol	-	0.2	1654	a, b
**57**	z-α-Santalol	-	0.1	1675	a, b
**58**	α-Bisabolol	-	0.2	1686	a, b
**59**	*epi*-β-Bisabolol	-	0.3	1672	a, b
**60**	z-*epi*-β-Santalol	-	0.1	1703	a, b
**61**	α-Vetivone	-	0.1	1823	a, b
**62**	n-Hexadecanol	-	0.1	1876	a, b

a = Kovat indices on a DB 5 column (1 µm thickness, 30.0 m length, 0.25 mm diameter); b = Mass fragmentation pattern.

**Table 2:** The antibacterial activity of *O. stamineus* and *F. deltoidea* oils and their active compounds against the oral pathogenic bacteria.

Samples	*O. stamineus* Essential Oil ^a^			*F. deltoidea* Essential Oil ^a^			*trans*-Caryophyllene ^a^			α-Humulene ^a^			Eugenol ^a^			Germacrene D ^a^			Amoxicillin ^b^	
Bacteria	MIC (mg/mL)	MBC (mg/mL)	Anti-Adhesion (mg/mL)	MIC (mg/mL)	MBC (mg/mL)	Anti-Adhesion (mg/mL)	MIC (mg/mL)	MBC (mg/mL)	Anti-Adhesion (mg/mL)	MIC (mg/mL)	MBC (mg/mL)	Anti-Adhesion (mg/mL)	MIC (mg/mL)	MBC (mg/mL)	Anti-Adhesion (mg/mL)	MIC (mg/mL)	MBC (mg/mL)	Anti-Adhesion (mg/mL)	MIC And MBC (mg/mL)	Anti-Adhesion (mg/mL)
*E. faecalis*	1.25	2.5	2.5	1.25	2.5	2.5	1.25	2.5	2.5	1.25	2.5	2.5	1.25	2.5	2.5	1.25	2.5	2.5	0.05	0.5
*S. mutans*	1.25	2.5	2.5	1.25	2.5	2.5	1.25	2.5	2.5	1.25	2.5	2.5	1.25	2.5	2.5	1.25	2.5	2.5	0.05	0.5
*S. mitis*	1.25	2.5	2.5	1.25	2.5	2.5	1.25	2.5	2.5	1.25	2.5	2.5	1.25	2.5	2.5	1.25	2.5	2.5	0.05	0.5
*S. salivarius*	1.25	2.5	2.5	1.25	2.5	2.5	0.63	1.25	1.25	0.63	1.25	1.25	1.25	2.5	2.5	1.25	2.5	2.5	0.05	0.5
*A. actinomy.*	1.25	2.5	2.5	0.63	1.25	1.25	0.63	1.25	1.25	0.63	1.25	1.25	1.25	2.5	2.5	1.25	2.5	2.5	0.05	0.5
*P. gingivalis*	1.25	2.5	2.5	0.63	1.25	1.25	0.63	1.25	1.25	0.63	1.25	1.25	1.25	2.5	2.5	1.25	2.5	2.5	0.05	0.5
*F. nucleatum*	1.25	2.5	2.5	0.63	1.25	1.25	0.63	1.25	1.25	0.63	1.25	1.25	1.25	2.5	2.5	1.25	2.5	2.5	0.05	0.5

^a^ concentrations ranging 0.04–5.0 mg/mL. ^b^ concentration at 0.05 mg/mL (positive control).

**Table 3:** Electron micrograph of the normal and altered morphological of the Gram-positive bacterial cells upon treatment with amoxicillin (positive control) and the *O. stamineus* and *F. deltoidea* oils. Enlargement: 10,000×.

Treatment	*E. faecalis*	*S. mutans*	*S. mitis*	*S. salivarius*
Untreated bacterial cell (normal)	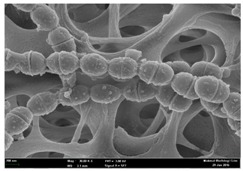	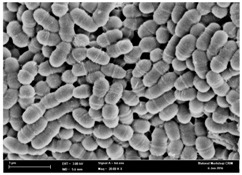	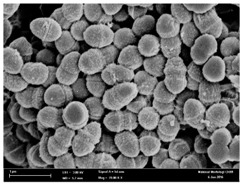	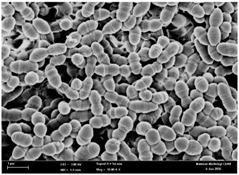
Positive control (treated with Amoxicillin) ^a^	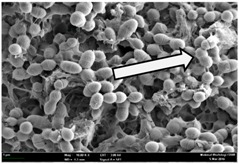	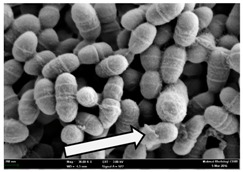	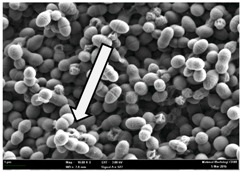	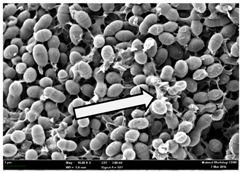
Bacterial cell treated with *O. stamineus* oil ^b^	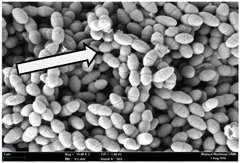	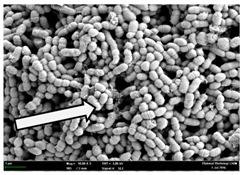	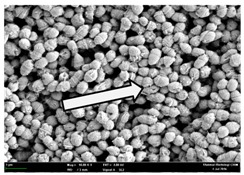	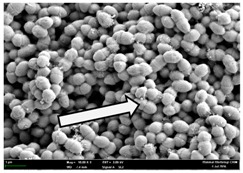
Bacterial cell treated with *F. deltoidea* oil ^c^	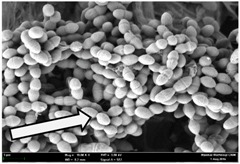	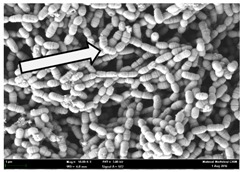	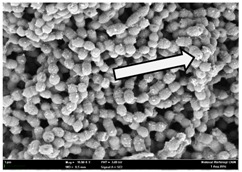	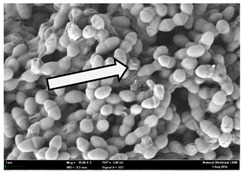

**^a^** All species of bacterial cells showed similar pattern of morphological changes due to the formation of crystallize structure (arrow) surrounding the cells that disrupt its structure and arrangement; **^b^** and **^c^** All species of bacterial cells showed similar pattern of morphological changes due to the presence of sticky and crystallize structure (arrow) surrounding the cells that disrupt its structure and arrangement.

**Table 4:** Electron micrograph of the normal and altered morphological of the Gram-negative bacterial cells upon treatment with amoxicillin (positive control) and the *O. stamineus* and *F. deltoidea* oils. Enlargement: 10,000×.

Treatment	*A. actinomycetemcomitans*	*P. gingivalis*	*F. nucleatum*
Untreated bacterial cell (normal)	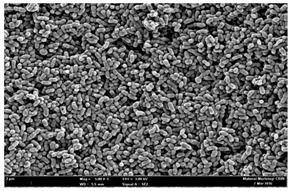	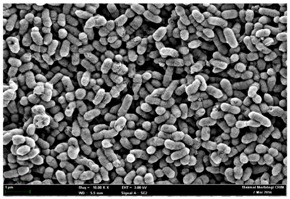	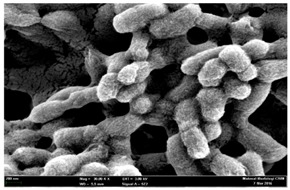
Positive control (treated with Amoxicillin) ^a^	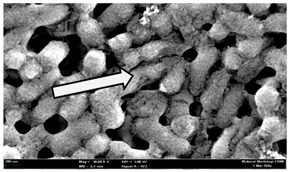	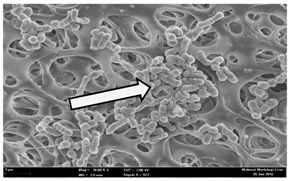	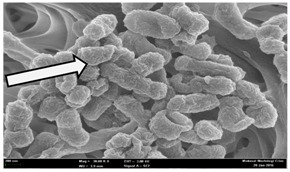
Bacterial cell treated with *O. stamineus* oil ^b^	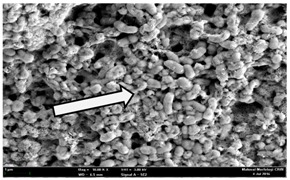	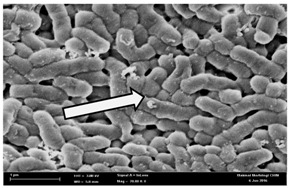	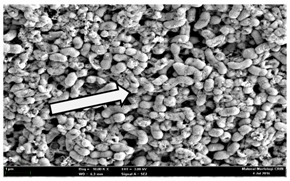
Bacterial cell treated with *F. deltoidea* oil ^c^	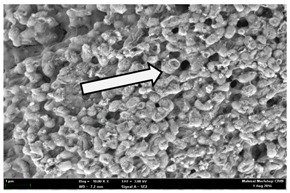	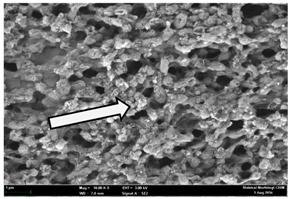	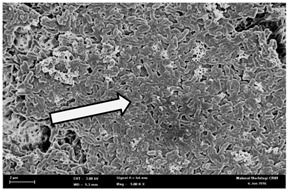

**^a^** All species of bacterial cells were pleomorphic (arrow) due to rupture and lysis; **^b^** Cells of *A. actinomycetemcomitans* and *F. nucleatum* were pleomorphic (arrow) due to rupture and lysis while cells of *P. gingivalis* collapsed and became transparent (arrow) due to lysis; **^c^** All species of bacterial cells were pleomorphic and became irregularly arranged (arrow) due to rupture and lysis.

**Table 5:** Synergistic activity of the essential oils and amoxicillin.

Bacteria	Essential Oils	MIC (mg/mL)				FIC Index ^e^	Activity
		Alone (Essential Oil) ^a^	Alone (Amoxicillin) ^b^	Combination (Essential Oil) ^c^	Combination (EO + Amoxicillin) ^d^		
*E. faecalis*	*O. stamineus*	1.25	0.05	0.625	0.0125	0.75	additive
	*F. deltoidea*	1.25	0.05	0.625	0.0125	0.75	additive
*S. mutans*	*O. stamineus*	1.25	0.05	0.625	0.0125	0.75	additive
	*F. deltoidea*	1.25	0.05	0.625	0.0125	0.75	additive
*S. mitis*	*O. stamineus*	1.25	0.05	0.625	0.0125	0.75	additive
	*F. deltoidea*	1.25	0.05	0.625	0.0125	0.75	additive
*S. salivarius*	*O. stamineus*	1.25	0.05	0.625	0.0125	0.75	additive
	*F. deltoidea*	0.63	0.05	0.625	0.0125	0.75	additive
*A. actinomycetemcomitans*	*O. stamineus*	1.25	0.05	0.625	0.0125	0.75	additive
	*F. deltoidea*	0.63	0.05	0.625	0.0125	0.75	additive
*P. gingivalis*	*O. stamineus*	1.25	0.05	0.625	0.0125	0.75	additive
	*F. deltoidea*	1.25	0.05	0.625	0.0125	0.75	additive
*F. nucleatum*	*O. stamineus*	1.25	0.05	0.625	0.0125	0.75	additive
	*F. deltoidea*	0.63	0.05	0.625	0.0125	0.75	additive

^a^ MIC value of essential oil. ^b^ MIC value of Amoxicillin. ^c^ MIC value of the combination of both essential oils. ^d^ MIC value of the combination of each essential oil with Amoxicillin. ^e^ fractional inhibitory concentration index.
